# Fatty Liver Due to Increased *de novo* Lipogenesis: Alterations in the Hepatic Peroxisomal Proteome

**DOI:** 10.3389/fcell.2019.00248

**Published:** 2019-10-25

**Authors:** Birgit Knebel, Pia Fahlbusch, Matthias Dille, Natalie Wahlers, Sonja Hartwig, Sylvia Jacob, Ulrike Kettel, Martina Schiller, Diran Herebian, Cornelia Koellmer, Stefan Lehr, Dirk Müller-Wieland, Jorg Kotzka

**Affiliations:** ^1^Leibniz Center for Diabetes Research, Institute of Clinical Biochemistry and Pathobiochemistry, German Diabetes Center at the Heinrich-Heine-University Duesseldorf, Düsseldorf, Germany; ^2^German Center for Diabetes Research (DZD), Partner Duesseldorf, Düsseldorf, Germany; ^3^Department of General Pediatrics, Neonatology and Pediatric Cardiology, Medical Faculty, University Children’s Hospital, Heinrich-Heine-University Düsseldorf, Düsseldorf, Germany; ^4^Department of Internal Medicine I, Clinical Research Centre, University Hospital Aachen, Aachen, Germany

**Keywords:** NAFLD, fatty liver, peroxisomes, label-free proteomic profiling, transcriptomics, lipidomics, SREBP-1c, DNL

## Abstract

In non-alcoholic fatty liver disease (NAFLD) caused by ectopic lipid accumulation, lipotoxicity is a crucial molecular risk factor. Mechanisms to eliminate lipid overflow can prevent the liver from functional complications. This may involve increased secretion of lipids or metabolic adaptation to ß-oxidation in lipid-degrading organelles such as mitochondria and peroxisomes. In addition to dietary factors, increased plasma fatty acid levels may be due to increased triglyceride synthesis, lipolysis, as well as *de novo* lipid synthesis (DNL) in the liver. In the present study, we investigated the impact of fatty liver caused by elevated DNL, in a transgenic mouse model with liver-specific overexpression of human sterol regulatory element-binding protein-1c (alb-SREBP-1c), on hepatic gene expression, on plasma lipids and especially on the proteome of peroxisomes by omics analyses, and we interpreted the results with knowledge-based analyses. In summary, the increased hepatic DNL is accompanied by marginal gene expression changes but massive changes in peroxisomal proteome. Furthermore, plasma phosphatidylcholine (PC) as well as lysoPC species were altered. Based on these observations, it can be speculated that the plasticity of organelles and their functionality may be directly affected by lipid overflow.

## Introduction

It is well known that mitochondria have a central role in lipid-degrading, but the metabolic function of peroxisomes is also becoming more important in order to counteract lipid accumulation in hepatocytes during metabolic stress (Unger et al., 2010; Fransen et al., 2012; Wanders, 2013; Knebel et al., 2015, 2018a; Wanders et al., 2015). Peroxisomes are specialized organelles involved in fatty acid ß-oxidation, whereas the substrate specificity is directed toward the ß-oxidation of very long chain- fatty acids and, by alpha-oxidation, also to branched-chain fatty acids.

In recent decades, the view of peroxisomes has changed from the previously considered role of degradation of branched or very long chain- fatty acids to fatty acids of medium chain length for further metabolic use in mitochondria. It is now clear that peroxisomes also act in specific anabolic processes, including the synthesis of bile acids for cholesterol clearance and produce ether lipids from lysophosphatidic precursors. The latter precursors were converted to special lipid species, i.e., plasmalogens (Lodhi and Semenkovich, 2014).

Another interesting development is the research of the biogenesis of peroxisomes. From the first theories, involving the cleavage and budding of a cellular ancestor peroxisome, it became clear that the *de novo* biogenesis of peroxisomes is a well-regulated assembly from diverse membrane structures. This process involves the budding of endoplasmatic reticulum (ER) and the import of cytosolically translated proteins, so that the unambiguous assignment of proteins to distinct organelle structure becomes more and more difficult (van der Zand et al., 2012; Schrader et al., 2013; Tabak et al., 2013). Moreover, mitochondria are also integrated in this flowing process (Dirkx et al., 2005; Tanaka et al., 2019). In addition, the close proximity of peroxisomes to lipid droplets led to an interaction of cellular structures to transfer lipids for degradation, but also the bilateral process is possible to exchange specific lipids (Binns et al., 2006; Bartz et al., 2007; Pu et al., 2011).

Recently, we were able to show that there is a significant overlap between mitochondrial and peroxisomal proteins in different stages of obesity and hyperphagia-induced NAFLD. In NAFLD, mitochondrial capacity increases, and this is a kind of dead end when maximum mitochondrial capacity is achieved. So, the increase in peroxisomes seems to be the emergency reserve to protect liver function. Thus, in a polygenic model of metabolic syndrome or monogenic leptin receptor defect, the activity of peroxisomes to support mitochondrial function initially increases (Knebel et al., 2015, 2018a).

To further narrow down the interaction of peroxisomes in the process of NAFLD we focused on the effect of solely hepatic lipid production. We intended to be independent of insulin sensitive adipose tissue derived lipolysis into the circulation, overall insulin resistance or affluent food consumption or specialized diets to introduce a NAFLD phenotype, as these procedures might account on secondary not defined effects on the liver physiology. So, in the present study, we used a mouse model, i.e., alb-SREBP-1c on C57Bl6 genetic background, with liver specific overexpression of the transcriptional active domain of the human transcription factor SREBP-1c, the master regulator of hepatic lipid synthesis (Knebel et al., 2012). In this model as main effect, *de novo* lipid synthesis (DNL) is constitutively activated in hepatocytes. Consequently, alb-SREBP-1c mice show a mild steatosis phenotype and selective hepatic insulin resistance due to activation of DNL and phenotypically this is accompanied by massive obesity and hepatic lipid accumulation with hepatic insulin resistance (Knebel et al., 2012; Jelenik et al., 2017).

To understand the role of peroxisomes in fatty liver due to genetically increased hepatic DNL we have (i) analyzed holistic hepatic gene expression, (ii) performed lipidomics, and (iii) determined the proteome of hepatic peroxisomes.

## Materials and Methods

### Animals

C57Bl6 (C57Bl6) and B6-TgN(alb-HA-SREBP-1cNT) (alb-SREBP-1c) (Knebel et al., 2012) mice were bred and maintained under standard conditions (12 h light/dark cycle; 22°C ± 1°C, 50% ± 5% humidity). The alb-SREBP-1c mice were backcrossed for more than 20 generations on C57Bl6 genetic background (Knebel et al., 2012) and C57Bl6 served as controls. At 6 weeks of age, male littermates of each genotype were kept under standardized conditions with free access to water and regular laboratory chow [13.7 mJ/kg: 53% carbohydrate, 36% protein, 11% fat (Ssniff, Soest, Germany)]. Mice were sacrificed by CO_2_ asphyxiation (7:00 a.m.) at 24 weeks of age. Mice were not fasted prior to sacrification. Blood samples were collected by left ventricular puncture, and livers were removed. The Animal Care Committee of the University Duesseldorf approved animal care and procedures (Approval#84-02.04.2015.A424; 02 April 2015).

### Animal Characterization

Phenotypical characterization, serum diagnostics of clinical measures, surrogate parameters of insulin resistance, and lipid profiling in serum, liver and adipose tissue by gas chromatography were performed as previously described (Kotzka et al., 2011; Knebel et al., 2012).

### Lipidomics

Serum free fatty acids, hepatic total fatty acids (TFA) content, and specific fractional compositions of FAs were determined by gas chromatography. fatty acids data were further used to calculate the Δ5-desaturase index (cC18:2/cC20:4), Δ6-desaturase index (cC18:2/cC18:3), Δ9-desaturase index (cC16:1/C16:0 or cC18:1/C18:0), DNL index (C16:0/cC18:2), and elongation index (C18:0/C16:0) as well as the sums of TFA, non-saturated fatty acids, monounsaturated fatty acids, saturated fatty acids, essential fatty acids (cC18:2 + cC18:3) or non-essential fatty acids (C16:0 + cC16:1 + C18:0 + cC18:1) (Cinci et al., 2000). Nomenclature used indicates Cx:y (x, number of carbons in the FA; y, number of double bonds in the fatty acids). Chemical residues [hydroxyl (OH), acyl (a), di-acyl (aa), and acyl-alkyl (ae)] are abbreviated accordingly. For metabolome analyses, plasma samples were rapidly frozen and stored at –80°C. Mass spectrometry for targeted metabolic profiling of glycerophospholipids and sphingolipids was performed using Biocrates methodology (Biocrates Life Sciences, Innsbruck, Austria). The limit of detection was determined for each metabolite from the signal to noise ratio. Metabolites were included in further analyses if values exceeded the respective limit of detection and could be detected in greater than 95% of the examined samples (Floegel et al., 2011; Knebel et al., 2016).

### Gene Expression Analyses

RNA extraction (Qiagen, Hilden, Germany) of snap frozen liver biopsies was performed as described (Knebel et al., 2018b). Genome wide expression analyses (*n* = 5 per genotype) were performed with 150 ng RNA according to the Ambion WT Expression Kit and the WT Terminal Labelling Kit (Thermo Fisher Scientific, Darmstadt, Germany). All protocol steps were monitored using an RNA 6000 nano kit (Agilent, Taufkirchen, Germany). Complementary RNA samples were hybridized to Mouse Gene 1.0 ST arrays and analyzed with a GeneChip scanner 3000 7G (GDAS 1.4 package, Affymetrix (Thermo Fisher Scientific, Darmstadt, Germany). Data were analyzed with Transcriptome Analysis Console^TM^ v4.01 (Applied Biosystems, Darmstadt, Germany) as described (Knebel et al., 2018b). Full datasets are available under accession number GSE132298^1^.

### Subcellular Fractionation

Enrichment of peroxisomes of a mouse liver via consecutive centrifugation approach [homogenate; Z1: 3000 × *g*, 15 min.; Z2: 17,000 × *g* 30 min.; density gradient: linear Optiprep^TM^ gradient (20–40%)] was performed as previously described (Hartwig et al., 2018). In iodixanol gradients, peroxisomes have the highest density of major subcellular organelles and can be safely isolated without detectable contamination by mitochondria or lysosomes, ER or Golgi membranes (Graham, 2002). All preparation steps were monitored by marker enzyme activities and electron microscopy (Supplementary Figures S3, S4). Mitochondrial copy number and monitoring of the preparation were determined as described (Hartwig et al., 2018).

### Proteomic Profiling of Peroxisomes

Protein profiling of the enriched peroxisomes was performed using LC-MS instrumentation consisting of an Ultimate 3000 separation liquid chromatography system (Thermo Fisher Scientific, Germering, Germany) combined with an EASY-spray ion source and Orbitrap Fusion^TM^ Lumos^TM^ Tribrid^TM^ mass spectrometer (Thermo Fisher Scientific), as previously described (Hartwig et al., 2013). Peptides were trapped on an Acclaim PepMap C18-LC-column (ID: 75 μm, 2 cm length; Thermo Fisher Scientific) and separated via an EASY-Spray C18 column (ES802; ID: 75 μm, 25 cm length; Thermo Fisher Scientific). Each LC-MS run lasted 120 min, and MS data were acquired with both data-dependent (DDA) and data-independent (DIA, 34 windows) MS/MS scan approaches. DDA runs were analyzed using Proteome Discoverer^TM^ 2.2 software (Thermo Fisher Scientific) and Sequest HT search (trypsin digestion, max. two miscleavages, 5–144 peptide length, max. 10 peptides per spectrum, carbamidomethylation as static and N-terminal acetylation/methionine oxidation as dynamic modifications) against the Swiss-Prot database [Mus musculus (TaxID = 10,090, version 2018–12)]. Percolator node-based peptide-spectrum-match (PSM) analysis was restricted to *q*-values with 0.01 (strict) and 0.05 (relaxed) false discovery rates (FDR). Proteins were filtered using parsimony set to 0.01/0.05 (strict/relaxed) FDRs. For quantification, DIA runs were analyzed via Spectronaut^TM^ Pulsar X 12.01 software (Biognosys, Zurich, Switzerland) set to standard parameter settings and using a self-performed spectral library based on DDA runs. For normalization, the proteomes were spiked with indexed Retention Time (iRT) standard.

### Statistical Analysis

Clinical values are presented as mean ± SD. Statistical analysis was performed with Student’s *t*-test calculated with Prism 7.4 (GraphPad Software Inc., San Diego, CA, United States), as indicated.

### Web-Based Functional Annotation

For functional annotation, web-based tools from public database sources were used: https://www.ncbi.nlm.nih.gov/, https://www.diabesityprot.org/, and IPA^®^ (Ingenuity^TM^, Qiagen, Hilden, Germany). To analyze the differential gene expression, fold change and *t*-test derived *p*-values of the comparisons, C57Bl6 vs. alb-SREBP-1c mice entered the analyses for IPA^®^. For analyses of the peroxisomal proteins, all proteins or values of change and *p*-values from PD analyses entered the analyses. Data were used for core analyses and comparison analyses. Pathways were generated from respective networks suggested by IPA^®^.

## Results

### Clinical Characterization

**FIGURE 1:**
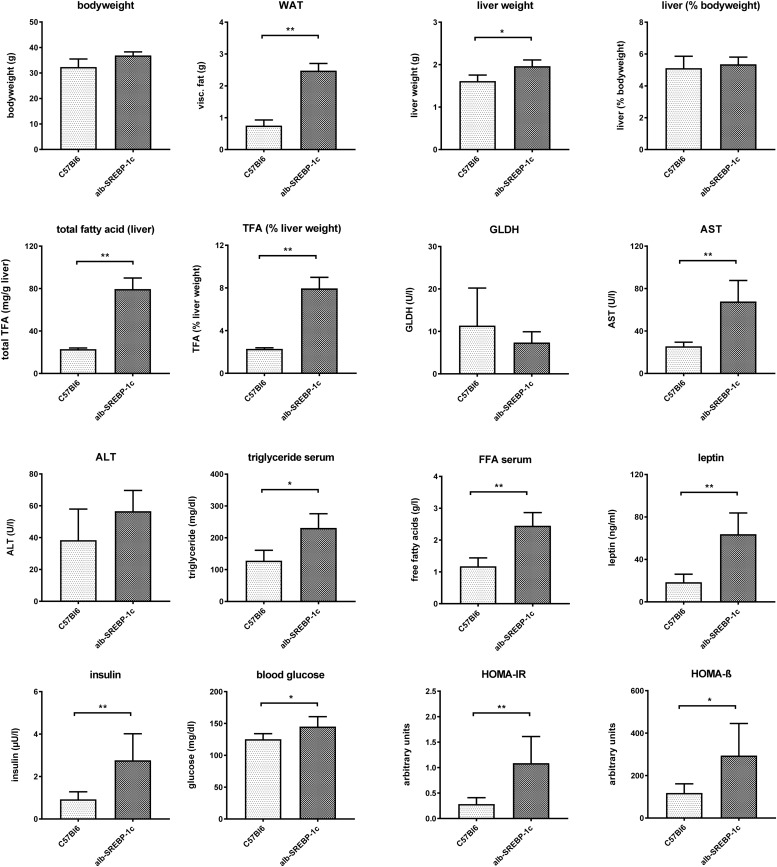
Metabolic characterization of C57Bl6 and of alb-SREBP-1c mice at the age of 24 weeks used in the study. Data are expressed as mean ± SD (*n* = 8 of each phenotype). ^∗^*p* < 0.05, ^∗∗^*p* < 0.01, by Student’s *t*-test. Abbreviations: ALT, alanine transaminase; AST, aspartate transaminase; FFA, free fatty acids; GLDH, glutamate dehydrogenase; HOMA-IR, Homeostatic model assessment of insulin resistance; HOMA-ß Homeostatic model assessment of ß-cell function (%); TFA, total fatty acids; WAT, white adipose tissue.

Alb-SREBP-1c mice display a mild hepatic steatosis due to increased DNL, which is caused by hepatic overexpression of the transcriptional active N-terminal domain of human SREBP-1c. The clinical characterization of the mice included in the present study is summarized in Figure 1.

Alb-SREBP-1c mice have increased body mass due to increased white fat tissue and liver weight. Liver weight is directly related to body weight. Here, the amount of TFA are increased per gram liver tissue resulting in an increased percentage share of lipids in the liver. The liver function markers remain unchanged at the age of 24 weeks, except significantly increased aspartate transaminase (AST). Free fatty acids, leptin and insulin are increased in serum. In addition, the blood sugar level is elevated, and the surrogate parameters for insulin resistance and ß-cell function indicate insulin resistance with compensatory insulin secretion (Figure 1). So, the mouse model used displays hepatic lipid accumulation due to increased DNL in the presence of selective hepatic insulin resistance.

### Gene Expression

First, we investigated the influence of the overexpression of SREBP-1c and thus activated hepatic DNL on the differential hepatic gene expression pattern, compared to controls. Holistic gene expression analyses showed a 1.4-fold expression difference, a total of 457 transcripts varied in abundance (163 increased in C57BL6 and 294 increased in alb-SREBP-1c) (Supplementary Table S1). Of these, 18 genes were direct and six genes were indirect downstream targets of SREBP-1c (Supplementary Figure S1A). The application of the complete knowledge-based SREBP-1 network identified about 50 indirect downstream targets of SREBP-1 in the dataset (Supplementary Figure S1B).

Knowledge-based analyses were further used to identify regulatory molecules that may be responsible for the changes in gene expression. Exemplified genes were validated by RT-PCR (Supplementary Figure S2). Here, SREBP-1 regulatory pathways, e.g., SREBP cleavage activating protein (SCAP) (*p*-value: 4.25E-09), and cholesterol metabolism with SREBF-2 (1.05E-08), cholesterol (1.69E-07), and cholic acid (4.97E-06) showed high significance (Supplementary Table S1). Next to SREBF-1 itself (9.10E-06), further central hepatic transcription factors such as HNF4A (9.32E-06), ligand-dependent nuclear receptors such as NR0B2 (1.01E-05), associated with Body Mass Index Quantitative Trait Locus 11 and related to the farnesoid X receptor pathway (FXR), or RXRA (1.40E-05) were enriched.

**FIGURE 2:**
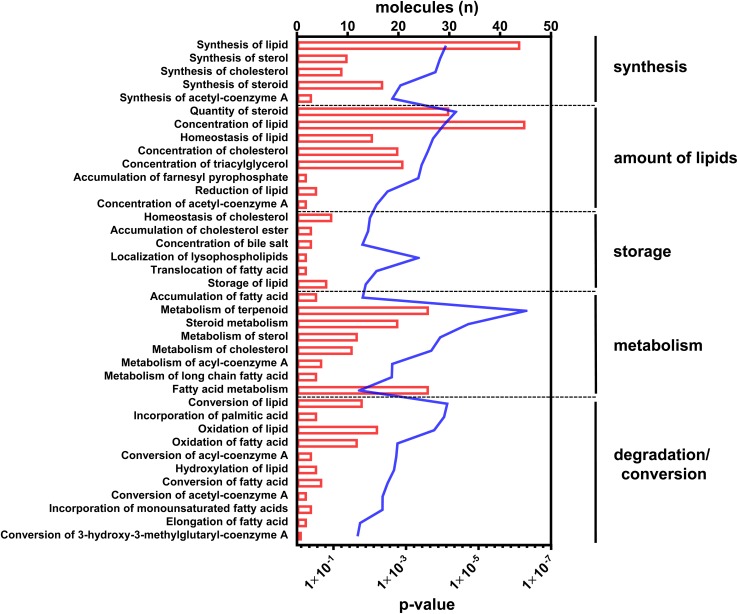
Canonical pathways annotated to lipid metabolism. Differential abundant transcripts in the comparisons of C57Bl6 and alb-SREBP-1c mice were subjected to knowledge-based analyses using IPA^®^. The bars indicate the number of differential abundant transcripts in the dataset associated to the respective pathway. The line indicates the significance of overlap to the respective pathway by Log10 transformed *p*-value. Analyses including statistics were performed in IPA^®^ using the Core expression analyses routine.

With regard to the functional readout of differential gene expression, 95 genes were associated with typical hepatic canonical pathways associated with metabolic disorders, for example, the sirtuin signaling pathway (1.66E-04), the EIF2 signaling pathway (1.26E-04), oxidative phosphorylation (9.12E-04), mitochondrial dysfunction (4.07E-04), LXR/RXR activation (5.13E-04), or cholesterol biosynthesis (1.86E-05). However, the majority of genes (*n* = 51) were assigned to different aspects of lipid metabolism (Figure 2 and Supplementary Table S2).

In accordance with these functional changes are further upstream regulators, such as RORA (1.88E-14), RORC (1.08E-13; with the differential expression itself being -1.616-fold), PPARA (5.56E-13), POR (1.50E-12), STAT5B (1.65E-09), glycerol 3-phosphate dehydrogenase 1 (GPD1) (2.00E-09), SLC25A13 (2.35E-09), NCOA2 (4.39E-06), SLC13A1 (9.11E-07), ATP7B transporter (2.44E-06), or NR1I3, a transcription factor associated with intrahepatic choleastis (2.88E-06). The latter is a RORA coactivator of G6PC expression, and thus regulates glucose metabolism. Furthermore, it is involved in the transcriptional activation of the glucocorticoid receptor (4.39E-06) and the peroxisomal ACOX1 (5.68E-06), both identified in these analyses (Supplementary Table S1).

**FIGURE 3:**
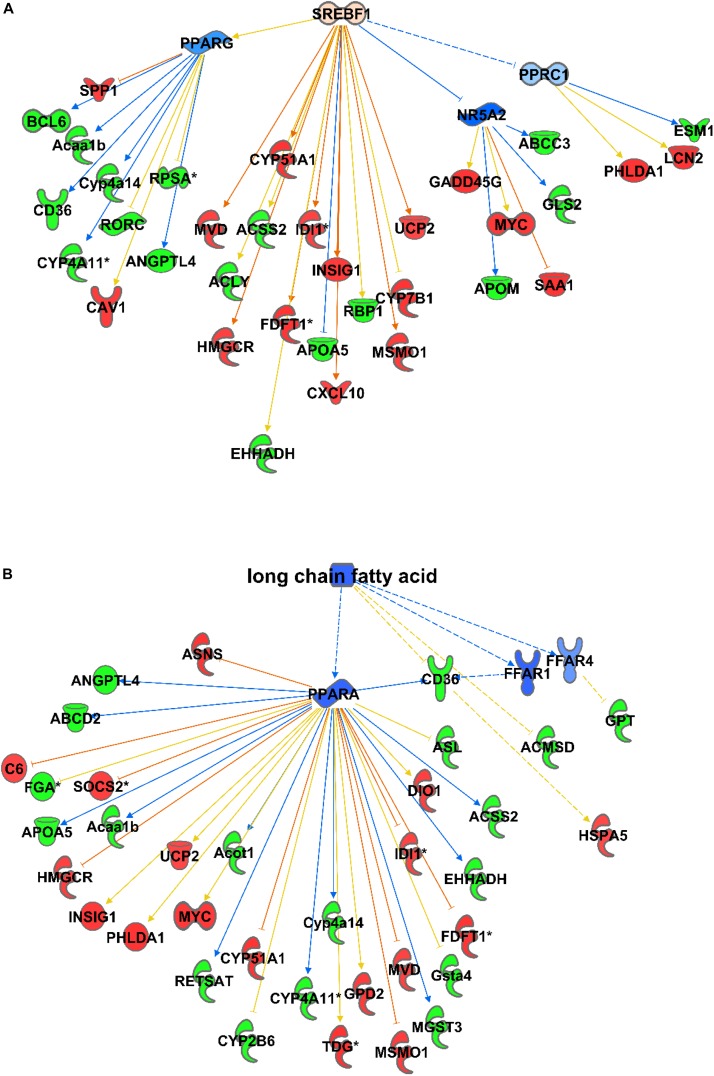
Causal networks for differences in gene expression. Differential abundant transcripts in the comparisons of C57Bl6 and alb-SREBP-1c mice were subjected to knowledge-based analyses using IPA^®^ to identify causal networks. SREBP-1 **(A)** and long chain fatty acids **(B)** were identified with high significance. Analyses were performed in IPA^®^ using the Core expression analyses routine. Color code, according to IPA^®^ analyses, for molecules: green: negative fold change (more abundant in C57Bl6); red: positive fold change (more abundant in alb-SREBP-1c), and for arrows: yellow: findings inconsistent with the state of the downstream molecule; blue: inhibition, consistent with the state of the downstream molecule; orange: increase, consistent with the state of the downstream molecule. Solid arrows indicate a direct interaction, and dotted arrows an indirect interaction, of connected molecules.

Consequently, among the causal activator networks that can play a role in differential gene expression as observed in C57Bl6 and alb-SREBP-1c mice is the SREBP-1 network with a *p*-value of 3.54E-08 (Figure 3A), Furthermore networks for SREBP-2 (3.36E-13), PPARA (2.4E-12), peroxisomal ACOX1 (8.63E-14), and GRK1 (2.48E-13) were involved. In addition, bioactive molecules such as apomin (3.36E-13), which binds FXR and regulates LDLR and HmgCoA reductase, or the lipid-lowering agent tiadenol (4.18E-14), can initiate a similar alteration in gene expression. In addition, metabolites such as long-chain fatty acids (8.40E-14) are identified as causal regulators that may cause alterations in gene expression networks (Figure 3B).

In summary, although the alterations in gene expression due to elevated hepatic DNL are rather marginal, the gene expression data point to lipid metabolism with the focus on long-chain fatty acids, but also on lipid secretion via cholesterol metabolism and bile acid pathways.

**FIGURE 4:**
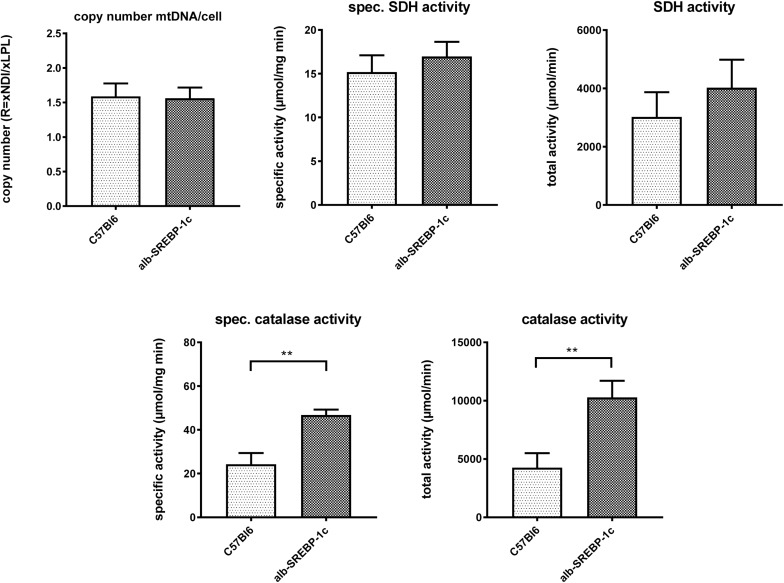
Mitochondrial DNA contend and marker enzyme activity of mitochondria and peroxisomes. The mtDNA content was determined in comparison to gDNA in C57Bl6 and alb-SREBP-1c mice (*n* = 20). Specific enzyme activities per mg liver tissue and total enzyme activities of mitochondrial succinate dehydrogenase (SDH) and peroxisomal catalase were determined in liver homogenates C57Bl6 and alb-SREBP-1c mice (*n* = 20) (n.s., not significant, ^∗∗^*p* < 0.01).

Interestingly, in liver lysates, the mitochondrial DNA copy count and the succinate dehydrogenase activity did not differ significantly within the models. However, peroxisomal catalase activity was increased in alb-SREBP-1c mice (Figure 4).

### Lipidomics

Peroxisomes are involved in lipid degradation, bile acid synthesis and synthesis of special lipids, and they are unique in the synthesis of ether lipids like alkyl ether phospholipids and plasmalogens (Kotzka et al., 2011). We performed a lipidomic screen to further narrow down the consequences of constantly increased DNL and hepatic insulin resistance.

**FIGURE 5:**
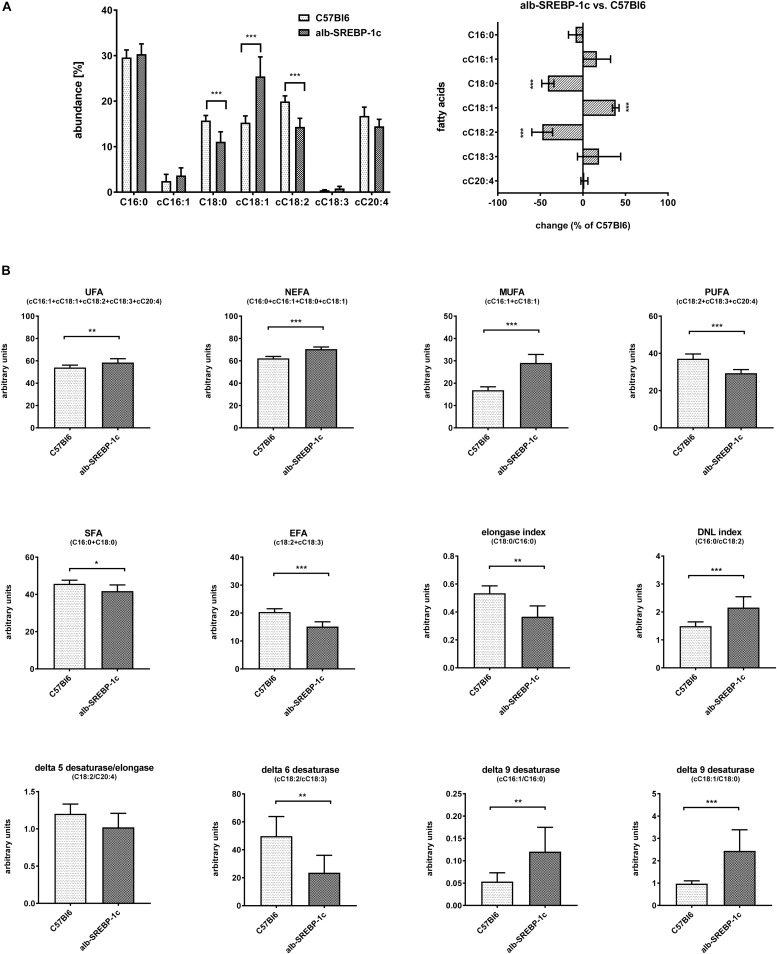
Hepatic lipid composition of C57Bl6 and alb-SREBP-1c mice at the age of 24 weeks. **(A)** Fractional composition of liver TFAs and %-change within C57Bl6 and alb-SREBP-1c mice. **(B)** From the hepatic lipid composition, the sums of non-saturated FA, non-essential FA (C16:0 + cC16:1 + C18:0 + cC18:1), monounsaturated FA, polyunsaturated FA, saturated FA, essential FA [cC18:2 + cC18:3) or elongation index (C18:0/C16:0)], *de novo* lipogenesis (DNL) index (C16:0/cC18:2), Δ5 desaturase index (cC18:2/cC20:4), Δ6 desaturase index (cC18:3/cC18:2), Δ9 desaturase index (cC16:1/C16:0), and Δ9 desaturase index (cC18:1/C18:0) were calculated. Data are expressed as mean ± SD (*n* = 8 of each genotype). ^∗^*p* < 0.05, ^∗∗^*p* < 0.01, ^∗∗∗^*p* < 0.01, by Student’s *t*-test. Abbreviations: DNL, *de novo* lipogenesis; EFA, essential fatty acids; MUFA, monounsaturated fatty acids; NEFA, non-essential fatty acids; PUFA, polyunsaturated fatty acids; SFA, saturated fatty acids; TFA, total fatty acids; UFA, unsaturated fatty acids.

Detailed analyses of serum lipids indicated no significant alterations within C57Bl6 and alb-SREBP-1c mice (Supplementary Figure S3). In contrast, lipid composition of hepatic TFA indicated an increase in cC18:1 and a decrease in C18:0 and cC18:2, in alb-SREBP-1c mice. In alb-SREBP-1c mice, the Δ9-desaturase activity on C16:1 and C18:1 as well as DNL were increased, and the Δ6-desaturase as well as elongase activity were decreased. This resulted in an increase in unsaturated fatty acids (UFA), monounsaturated fatty acids (MUFA), and non-essential fatty acids (NEFA) and a decrease in saturated fatty acids (SFA) and polyunsaturated fatty acids (PUFA), in alb-SREBP-1c mice (Figure 5).

**FIGURE 6:**
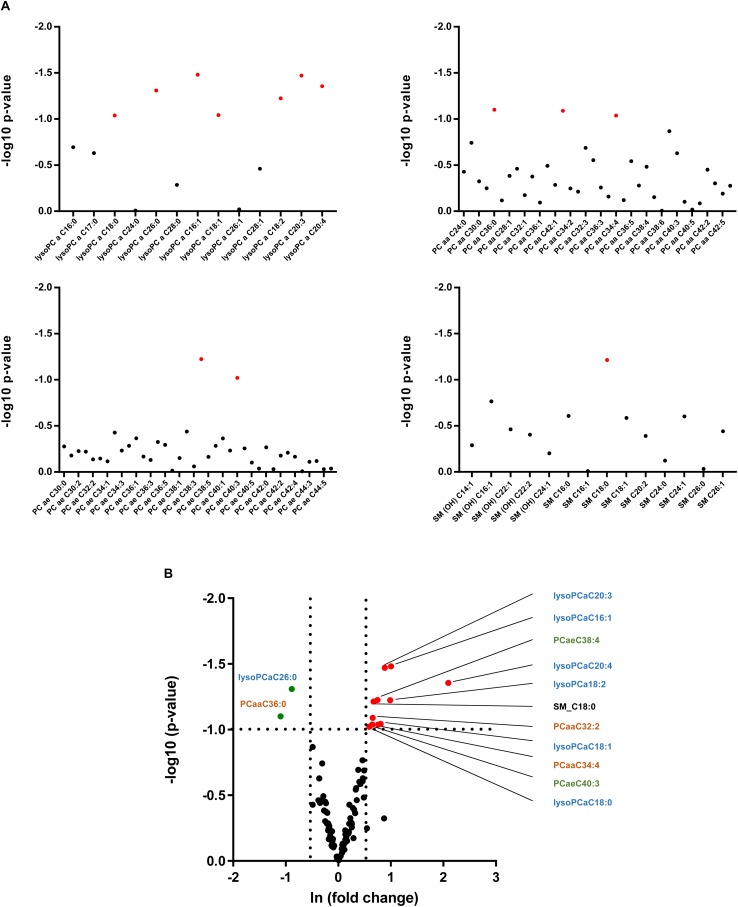
Lipid species in the serum of C57Bl6 and alb-SREBP-1 mice. **(A)** Alterations in individual lipid species in the group of phosphatidylcholines (PC), i.e., lysoPCs, PCaa, PCae, and sphingomyelins are indicated by log 10 *p*-value. Red dots indicate significant altered lipid species. **(B)** Differential abundance of lipid species. The fold change [(ln(fold change)] of lipid concentration was plotted against the significance (log 10 *p*-value). Red dots mark lipid species with >1.5-fold change in alb-SREBP-1c mice, green dots indicate lipid species with >1.5-fold difference in C57Bl6 mice. A *p*-value of <0.05 (Students’ *t*-test) was accepted as significant. Chemical residues [hydroxyl (OH), acyl (a), di-acyl (aa), and acyl-alkyl (ae)] are abbreviated accordingly. Nomenclature further indicates Cx:y (x, number of carbons in the fatty acid; y, number of double bonds in the fatty acid).

As alterations in hepatic lipid composition were not reflected in circulating serum free fatty acids, we performed a more comprehensive serum lipidomic approach to determine special lipid class including lysophosphatidyl choline (lysoPC), acyl-acyl-phosphatidyl choline (PCaa), acyl-alkyl-phosphatidyl choline (PCae) and sphingomyelin (SM) lipid species (Figure 6A). In alb-SREBP-1c mice, we determined a decrease in lysoPCaC26:0 as well as PCaaC36:0, and an increase in SM_C18:0, PCaa32:2 as well as PCaaC34:4 and PCaeC38:4 as well as PCaeC40:3 and various lysoPCs, i.e., –aC16:1, –aC18:0, –aC18:1, –aC18:2, –cC20:3, and –aC20:4 (Figure 6B). The alterations especially of lysoPC species further focused on the role of peroxisomes.

### Peroxisomal Proteome

The alterations in gene expression, marker enzymes for organelle function, and lipid metabolism observed point toward peroxisomal function or organization. Therefore, we focused on the analyses of peroxisomal proteomes in livers of control mice and mice with increased hepatic lipid accumulation.

For the preparation of peroxisomes, we used our standard protocol (Graham, 2002; Knebel et al., 2015, 2018a). The enrichment of peroxisomes in the gradient was monitored by cell organelle specific marker enzyme assays (Supplementary Figure S4). This peroxisomal enrichment was confirmed by electron microscopy, indicating enriched peroxisomal structures, without visible mitochondrial structures (Supplementary Figure S4). Deduced from marker enzyme assays the enrichment of peroxisomes didn’t differ between C57Bl6 and alb-SREBP-1c mice (Supplementary Figure S5). High resolution mass spectrometry analyses identified a total of 2,295 unique proteins in the peroxisomal protein fractions. Of these, 1,053 proteins were overrepresented whereas 944 proteins were underrepresented in alb-SREBP-1c compared to C57Bl6 mice (Supplementary Table S3). In contrast to the relative low number of differentially regulated genes, this number is surprising high and reflects the largest difference observed in the alb-SREBP-1c model compared to C57Bl6 model up to now (Knebel et al., 2012; Jelenik et al., 2017).

**FIGURE 7:**
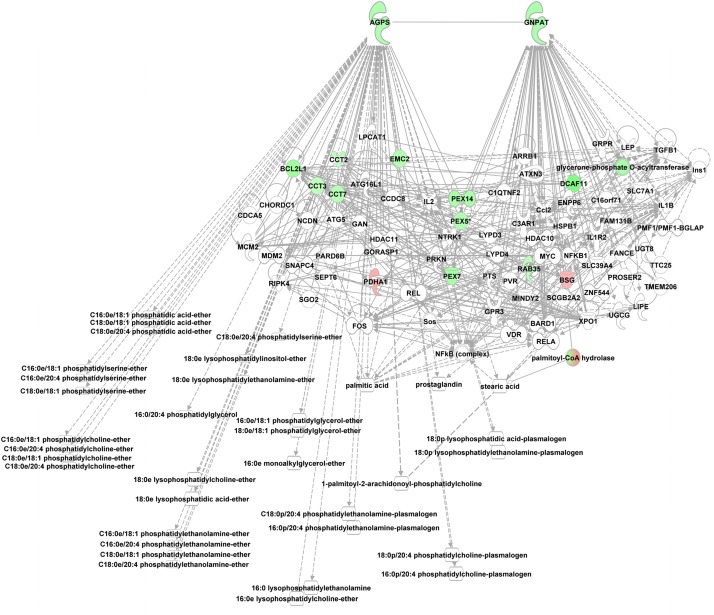
Knowledge-based analyses enzymes essential in ether lipid analyses in peroxisomal protein fractions. All proteins identified in peroxisomal fractions of C57Bl6 mice and alb-SREBP-1c mice were subjected to IPA^®^ Core analyses. The networks deduced for upstream regulator molecules glyceronephosphate O-acyltransferase (GNPAT) and alkylglycerone phosphate synthase (AGPS) are shown. Color code, according to IPA analyses, for molecules: green, overrepresented in alb-SREBP-1c (negative fold change in dataset); red: overrepresented in C57Bl6 (positive fold change in dataset). Solid arrows indicate a direct interaction, and dotted arrows an indirect interaction, of connected molecules.

Phosphoetherlipid synthesis requires peroxisome and ER associated enzymes, i.e., glycerol phosphate O-acyltransferase (GNPAT), essential for phosphoether lipid synthesis and alkylglycerone phosphate synthase (AGPS) which catalyzes the exchange of an acyl for a long-chain alkyl group and the formation of the ether bond in the biosynthesis of ether phospholipids using e.g., lysoPCs as substrate. Both enzymes were enriched in the alb-SREBP-1c peroxisomal protein fractions compared to controls. Furthermore, interacting proteins like CCT or PEX proteins were also enriched (Figure 7).

All identified proteins from peroxisomal fractions (Supplementary Table S4) were used in knowledge-based analyses to analyze for the overall accumulation of canonical pathways.

Examples with the highest *p*-values were: various aspects cholesterol biosynthesis (2.57E-10-6.31E-14) or lipid metabolism [stearate biosynthesis (3.98E-20), fatty acid β-oxidation (1.26E-23)], or the superpathway of melatonin degradation (1.99E-10), nicotine degradation (3.98E-12). Nuclear receptor LXR/RXR activation (1.26E-12) or FXR/RXR activation (7.94E-15), as well as LPS/IL-1-mediated inhibition of RXR function (3.16E-18), were also involved. Furthermore, proteins annotated to estrogen biosynthesis (6.31E-13), TCA cycle (1.58E-14), mTOR signaling (1.0E-13), amino acid degradation of valine (3.16E-15), isoleucine (2.51E-11) or tryptophan (2.52E-11), or stress and redox response, e.g., acute phase response (3.16E-12), EIF2 signaling (6.31E-35) or eIF4 and p70S6K signaling (1.0E-12), or NRF2-mediated oxidative stress response (2.51E-14). Pathways involving membrane systems like actin nucleation (5.01E-11), actin cytoskeleton signaling (3.16E-11), cathrin-mediated endocytosis signaling (1.99E-11), epithelial adherens junction signaling (3.16E-13), or remodeling of epithelial adherens junctions (1E-16) might indicate the role of peroxisomes in the cellular endomembrane systems. In regard to the downstream effects of peroxisomal proteins, major functional overlaps were assigned to sirtuin signaling pathway (3.16E-37), oxidative phosphorylation (5.01E-62) and mitochondrial dysfunction (5.01E-75), or fatty acids metabolism (1.55E-04). So, next to processes involved in lipid or energy metabolism, lipid degradation and cholesterol clearance, a further functional focus was on membrane system compositions and dynamics.

**FIGURE 8:**
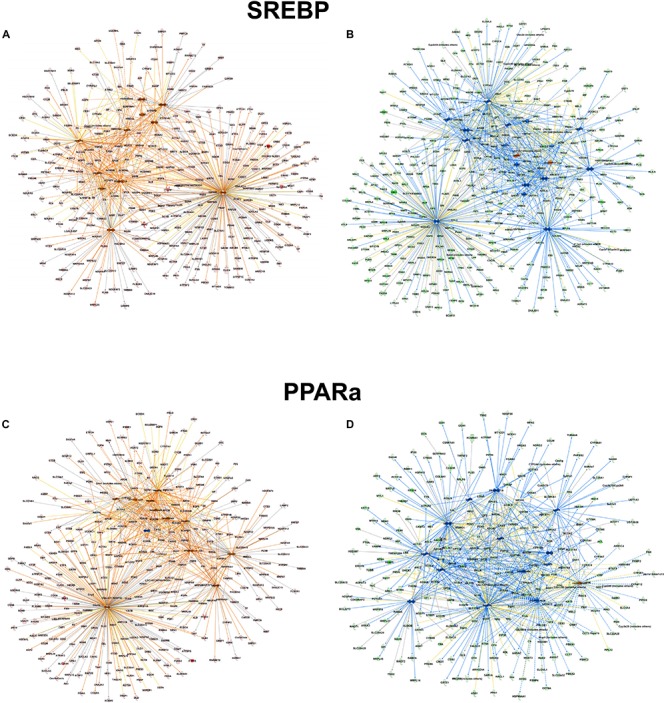
Upstream regulator molecules in peroxisomal proteins. All proteins identified in the peroxisomal fraction of C57Bl6 mice and alb-SREBP-1c mice were separated, according to overrepresentation in the genotypes, and subjected to IPA^®^ Core analyses. Upstream regulator molecule networks are shown for SREBP in C57Bl6 overrepresented proteins **(A)**, or in alb-SREBP-1c overrepresented proteins **(B)** and for PPARa in C57Bl6 overrepresented proteins **(C)**, or in alb-SREBP-1c overrepresented proteins **(D)**. Hub molecules for SREBF1 in C57Bl6 mice were cholesterol, SCAP, Insulin, SREBF2, PPARG, PPARA, HIF1A, FOXO1, PPARGC1A, TP53, and RXRA. Hub proteins/molecules for SREBF1 in alb-SREBP-1c mice were SCAP, cholesterol, fatty acid, Ins1, PPARD, NR1H3, PPARG, PPARA, NR0B2, CEBPB, RXRA, SREBF2, NR5A2, NFE2L2, TP53, and PPARGC1A. Hub proteins/molecules for PPARA in C57Bl6 mice were bezafibrate, pirinixic acid, fenofibrate, NR1H4, PPARG, PPARD, NRIP1, RXRA, FOXO1, SREBF1, TP53, PPARGC1A, and NCOA2. Hub proteins/molecules for PPARA in alb-SREBP-1c mice were ciprofibrate, fenofibrate, pirinixic acid, NR1H4, PPARG, PPARD, MED1, NCOA2, NR1H3, FOXO1, RXRA, SREBF1, NR1I3, THRB, and PPARGC1A. Color code, according to IPA^®^ analyses, for molecules: green: overrepresented in alb-SREBP-1c (negative fold change in dataset); red: overrepresented in C57Bl6 (positive fold change in dataset), and for arrows: yellow: findings inconsistent with the state of the downstream molecule; blue: inhibition, consistent with the state of the downstream molecule; orange: increase, consistent with the state of the downstream molecule. Solid arrows indicate a direct interaction, and dotted arrows an indirect interaction, of connected molecules.

However, focusing on the differential abundance in peroxisomal protein patterns in the mouse models, upstream regulators deduced from the specific datasets were addressed differentially (Supplementary Tables S5–S7). For example in the alb-SREBP-1c overrepresented peroxisomal dataset (Supplementary Table S6), SREBP-1 as an upstream activator had a negative activation z-score of -4 (*p*-value 1.95 E-15), indicating inhibition, whereas in C57BL6 overrepresented data (Supplementary Table S7), a 4.4-fold activation z-score was determined, although with lower significance (*p*-value 2.15 E-10). Additionally, there are common but also specific hub proteins or metabolites in the comparisons. In C57Bl6 mice, HIF1A and FOXO1 were identified as SREBP-1 nodal proteins whereas fatty acids, PPARD, NR1H3, NROB2, CEBP, NR5A2, and NFE2l2-targeted SREBP actions were present specifically in the alb-SREBP-1c overrepresented dataset. The pattern of the upstream activator PPARA in the datasets is comparable. In data overrepresented in alb-SREBP-1c mice, a z-score of -2.91 with a *p*-value of 1.95 E-46, and in C57Bl6 overrepresented data, a z-score of 3.096 with a *p*-value of 1.45 E-19 was found for PPARA as an upstream activator. Specific hub molecules in the C57Bl6 overrepresented data were bezafibrate, NRIP1 and TP53. However, a different fibrate, i.e., ciprofibrate was a nodal upstream regulator in the alb-SREBP-1c overrepresented dataset. This might indicate a differential pharmacogenetics of the fibrates, although tagging the same PPARA molecule as orphan receptor. Furthermore, MED1, NR1H3/LXR NR1| 3 and THRB were unique nodal points in the alb-SREBP-1c overrepresented data (Figure 8 and Supplementary Table S8).

**FIGURE 9:**
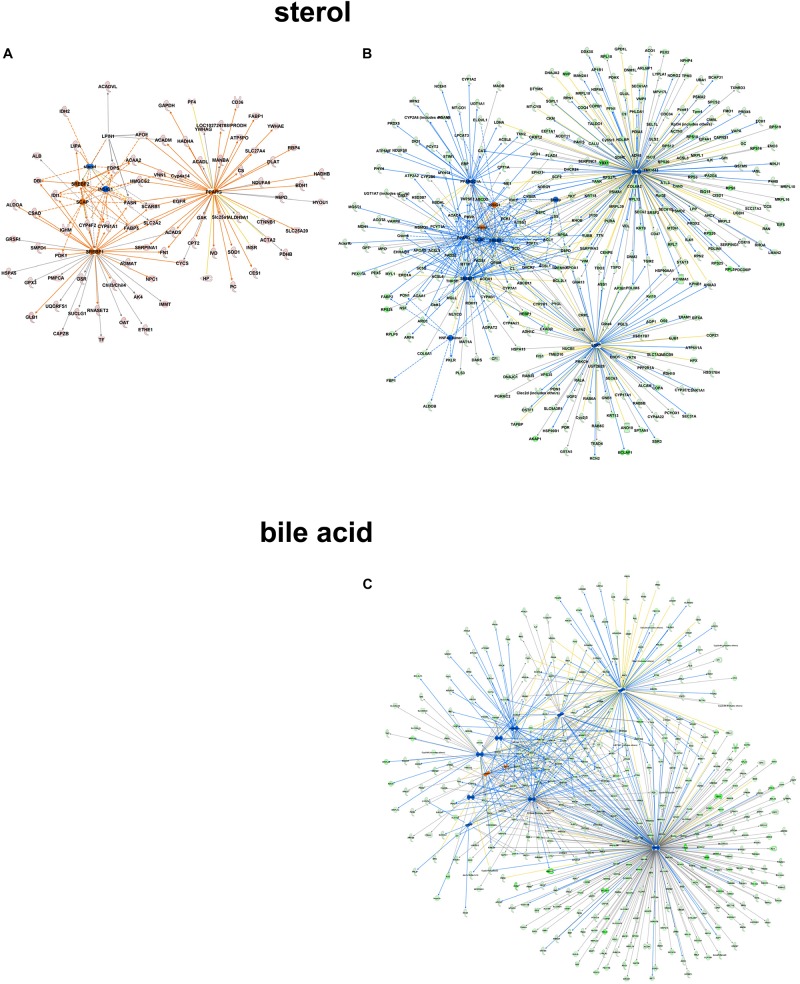
Upstream regulator metabolites in peroxisomal proteins. All proteins identified in peroxisomal fractions of C57Bl6 mice and alb-SREBP-1c mice were separated, according to overrepresentation in the genotypes, and subjected to IPA^®^ Core analyses. Upstream regulator molecule networks are shown for sterol in C57Bl6 overrepresented proteins **(A)**, or in alb-SREBP-1c overrepresented proteins **(B)**, and for bile acid in alb-SREBP-1c overrepresented proteins **(C)**. Upstream regulator molecule networks are shown for sterol **(A)** and bile acid **(B)**. Hub proteins for sterol in C57Bl6 were PPARG, SCAP, LPIN1, INSIG1, SREBF1, and SREBF2. Hub proteins for sterol in alb-SREBP-1c were: PPARG, SCAP, INSIG1, SREBF1, SREBF2, HNF4α dimer, ESR1, TP53, and PPARGC1A. Hub proteins for bile acid in alb-SREBP-1c were: PPARA, FOXO1, FGF19, NR1H4, NR0B2, HNF4A, MLXIPL, NR5A2, FOXA2, RXRA, HNF1A, and PPARGC1A. Color code, according to IPA^®^ analyses, for molecules: green: overrepresented in alb-SREBP-1c (negative fold change in dataset); red: overrepresented in C57Bl6 (positive fold change in dataset), and for arrows: yellow: findings inconsistent with the state of the downstream molecule; blue: inhibition, consistent with the state of the downstream molecule; orange: increase, consistent with the state of the downstream molecule. Solid arrows indicate a direct interaction, and dotted arrows an indirect interaction, of connected molecules.

Focusing on the role of metabolites like sterol as upstream regulatory molecules, only a limited number of molecules were overrepresented in the C57Bl6 dataset (z-score -1.486, *p*-value 1.01-E3), with LPIN1 as a unique nodal gene. In the alb-SREBP-1c overrepresented dataset, a positive regulatory effect was predicted (z-score 1.791, *p*-value 3.25 E-10). Here, more proteins with higher significance were involved, and HNF4a, EsR1, TP53, and PPARGC1A were specifically addressed. In contrast, e.g., bile acid responsive proteins were only present in the alb-SREBP-1c overrepresented data (z-score 0.339, *p*-value 7.15 E-3) with nodal points PPARa, FOXO1, FGF19, NR1H4, NROB2, HNF4a, ChREBP (MLXIPL), NR5A2, FOXA2, RXRA, HNF1A, and PPARGC1A (Figure 9 and Supplementary Table S9). The results indicate a differential response pattern depending on the cellular physiology as caused by hepatic lipid accumulation in the models investigated, to either a central regulatory molecule, or the presence of a metabolite.

### Functional Readout of Differential Peroxisomal Proteome

**FIGURE 10:**
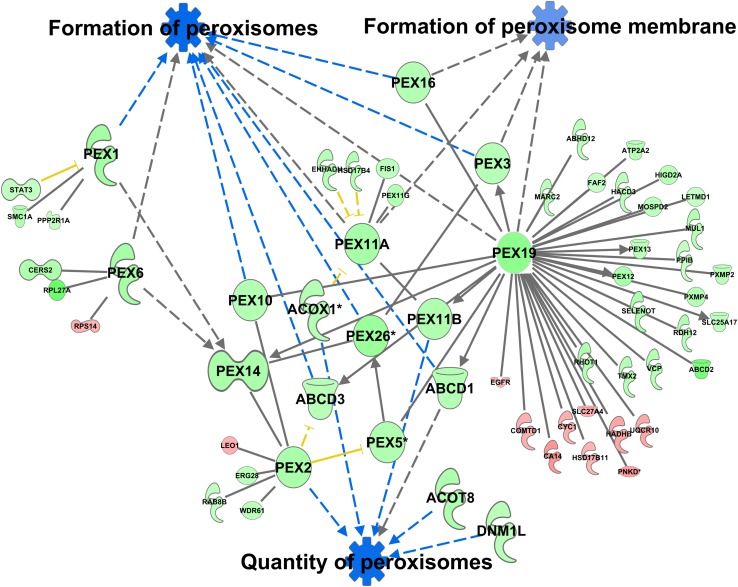
Differential peroxisomal formation deduced from the peroxisomal proteome. Proteins with at least 1.5-fold difference in abundance identified in peroxisomal fractions of C57Bl6 mice and alb-SREBP-1c mice were subjected to IPA^®^ Core analyses. Peroxisomal formation and associated proteins were identified as the downstream function with highest significance. The datasets of peroxisomal proteins enriched in either C57Bl6 or alb-SREBP-1c were screened for interacting proteins of the peroxisomal formation node. Color code, according to IPA^®^ analyses, for molecules: green: overrepresented in alb-SREBP-1c (negative fold change in dataset); red: overrepresented in C57Bl6 (positive fold change in dataset), and for arrows: yellow: findings inconsistent with the state of the downstream molecule; blue: inhibition, consistent with the state of the downstream molecule. Solid arrows indicate a direct interaction, and dotted arrows an indirect interaction, of connected molecules. Molecules not in direct connection to the downstream functions are shown in reduced size.

A total of 370 proteins in the peroxisomal protein fraction showed at least a 1.5-fold difference (111 more abundant in C57Bl6, 259 more abundant in alb-SREBP-1c). Knowledge-based analyses identified the formation of peroxisomes and related pathways, like the formation of peroxisomal membrane, and the quantity of peroxisomes as the downstream function with the highest *p*-value (2.82E-07) (Supplementary Table S10), with 6 central proteins to this function (PEX1, PEX2, PEX6, PEX11A, PEX19, and PEX26) (Figure 10).

Increasing the interacting protein network of these proteins with overrepresented proteins in alb-SREBP-1c, a total of 44 proteins with 64 new interactions were added to this nodal pathway. In contrast from C57Bl6 overrepresented proteins only 11 proteins and interactions were added. The latter showed no direct relation to the downstream functions, but were connected via central hub proteins e.g., PEX19 with known function in import of peroxisomal membrane proteins and the *de novo* formation of peroxisomes.

## Discussion

Ectopic hepatic lipid accumulation, such as in NAFLD, is a severe health burden (Hajra and Das, 1996). Mechanisms leading to the reduction of lipid accumulation are therefore of particular interest to maintain liver physiology and to avoid lipotoxicity and thus the long-term development of hepatic lipid accumulation into cirrhosis. In this study, we investigated the processes of ectopic hepatic lipid accumulation in a mouse model with genetically elevated DNL. In this observational study, we show that: (i) the change in hepatic gene regulation is low and no unexpected novel signaling pathways are identified in sufficient significance, (ii) the changes in hepatic TFA are reflected in serum phospholipid species, and (iii) alterations of the proteome of the peroxisomal protein fraction.

The basic-helix-loop-helix-leucine zipper family transcription factor SREBP-1c is involved in the regulation of genes involved in lipid and cholesterol synthesis. Although the pathogenesis of NAFLD is not yet clear in detail, the regulation of the transcription factor SREBP-1c is one central event. SREBP-1 proteins are tightly regulated on various levels: (i) on direct transcriptional level, (ii) by a complex regulatory mechanism involving coordinated proteolytic release of the transcriptional active domain from a precursor molecule, (iii) on post-translational modification to regulate its transcriptional activity and stability, and last not least (iv) by homo- and heterodimerization for DNA interaction with isoform specific impact on gene regulation. Each step of this orchestrated regulation integrates information of the metabolic status of a cell to the transactivation of SREBP-1, with SREBP-1c being the predominant isoform in lipid metabolism (Brown and Goldstein, 2009; Ferre and Foufelle, 2010; Shimano and Sato, 2017; Younossi, 2019). The specific activation of hepatic DNL in these animals is induced by liver specific overexpression of the N-terminal transcriptionally active domain of human sterol regulatory element-binding protein (SREBP)-1c driven by albumin promoter (Knebel et al., 2012). This circumventing the need of all regulatory steps, i.e., *de novo* transcription and especially the highly regulated release of the mature transcription factor. The investigated transgenic mouse model shows a mild fatty liver, hepatic insulin resistance with compensatory increased ß-cell function, and massive obesity but no signs of inflammation and metabolically healthy adipose tissue (Knebel et al., 2012, 2019; Jelenik et al., 2017).

However, the liver-specific overexpression of SREBP-1c leads to a comparatively small change in hepatic gene expression, analogous to the isoform SREBP-1a (Knebel et al., 2018b). On the one hand, this is not unexpected as only one protein is altered; on the other hand, it seems surprising that this minimal change in gene expression can be sufficient to initiate the first steps of fatty liver development.

The gene expression analyses here showed no unexpected signaling or metabolic pathways. The central change in gene expression is in the area of lipid metabolism, central molecules of liver metabolism and the regulation of SREBP such as SCAP, or the synthesis of bile acid to facilitate cholesterol clearance.

The mouse model used overexpresses the human N-terminal domain of SREBP-1c (Knebel et al., 2012). Since the expression of the intrinsic SREBP-1c molecule is not increased (Knebel et al., 2012), a feedback loop might be active to compensate for the overrepresentation of the human SREBP molecule. One could speculate that the permanently genetically activated DNL leads to a general regulatory compensation in the hepatocytes. Alternatively, metabolic adaptation may occur. Consequently, cholesterol, bile acid and especially long-chain fatty acids are superior factors that lead to the patterns of altered gene expressions observed.

*De novo* lipogenesis produces C16:0 and C18:0, which are further elongated or desaturated during the process, thereby stimulating the production of monounsaturated fatty acids such as cC16:1 and cC18:1. Also, the hepatic overexpression of SREBP-1c increases DNL and activates the expression and activity of stearoyl CoA desaturase (SCD), elongases and desaturases (Ferre and Foufelle, 2010; Knebel et al., 2012). This is reflected in the hepatic lipid indices observed here.

The analysis of lipids in the liver shows specific differences which are not reflected in the circulating species of plasma lipids. However, detailed plasma lipid analyses showed that elevated DNL is accompanied by changes in glycerolipid and phosphatidylcholine (PC) patterns, especially in lysoPCs. PCs are major components of plasma phospholipids and thus of lipoproteins. In humans, more than 76% of total glycerophospholipids consists of PC and lysoPCs (Quehenberger et al., 2010; Yang et al., 2018). PCs were mainly formed with four types of fatty acids (C16:0, C18:0, cC18:1, cC18:2) (Phillips and Dodge, 2018), and the composition interferes with the function. Thus, the PCs C16:0/cC18:1 and C18:0/cC18:1 are endogenous, physiologically relevant PPARα ligands, which regulate glucose homeostasis or lipid metabolism (Chakravarthy et al., 2009; Liu et al., 2013). Other PCs with C16:0/cC20:4 or C16:0/cC18:2 composition activate AKT signaling (Koeberle et al., 2013). Furthermore, high density-lipoprotein particles containing the PC C16:0/cC18:1 were the most effective acceptor of free cholesterol to facilitate efflux (Schwendeman et al., 2015).

In addition to PCs, in particular lysoPCs show differences depending on DNL activation in the models investigated in the study. The most common lysoPC species in normo-lipidemic plasma is lysoPC 16:0. Its content decreases in hyper-lipidemic conditions with simultaneous increases in free fatty acids, short chain saturated fatty acids, diacylglycerols, triacylglycerols and PCs (Rai and Bhatnagar, 2017). LysoPC 16:0 also regulates the peroxisome proliferator-activated receptor alpha, and thus the uptake and oxidation of fatty acids (Phillips and Dodge, 2018). Consistent with the increased DNL, in the alb-SREBP-1c model not C16:0 but the C16:0 desaturation product C16:1, and the elongation products C18:0 and cC18:1 occurs as major fatty acid in lysoPCs. cC18:2 and longer-chain fatty acids are more abundant in lysoPCs compared to controls. In this context is of interesting note that the peroxisomal proteins GNPAT and AGPS, initializing enzymes of phosphoetherlipid synthesis, are increased in the peroxisomal protein fraction of alb-SREBP-1c mice.

Another finding of the study is the increased content of initially mitochondrial-annotated proteins, and core downstream functions such as oxidative phosphorylation, in the increased DNL state. Peroxisomes are involved in specific lipid degradation processes, including several steps of cholesterol synthesis to its secretion via bile acids. Under normal metabolic conditions, the percentage of peroxisomal ß-oxidation of e.g., palmitate is apparently between 10 and 30% (Kondrup and Lazarow, 1985). However, it can increase significantly depending on the available substrates or the metabolic state. Peroxisomes are therefore highly flexible and have been associated with various metabolic diseases such as diabetes and fatty liver (Wanders, 2013; Knebel et al., 2015, 2018a; Wanders et al., 2015). Recently, we have shown a significant overlap between mitochondrial and peroxisomal proteins in different stages of obesity and hyperphagia-induced NAFLD, and the increase in peroxisomes seems to be the emergency reserve to protect liver function (Knebel et al., 2015, 2018a). In these models central hepatic regulators affecting, e.g., PPARa or HN4a axis and increased SREBP-1c expression were involved. However, according to the severity of the phenotype investigated, an orchestrated alteration of activated downstream molecules occurs that modulate the effect on the peroxisomes from functional activation to decay (Knebel et al., 2015, 2018a,2018b).

It is well known that peroxisomes share enzymes, e.g., for ß-oxidation, with mitochondria, and overlapping proteoms of the organelles were described (Hartwig et al., 2013; Schrader et al., 2013; Wanders, 2013; Knebel et al., 2015, 2018a,b; Fransen et al., 2017). In this context, the study of Krahmer et al. (2018) is also of interest, who have elegantly shown in detailed mass spectrometric analyses that in stages of diet-induced hepatic lipid accumulation the size fractionation pattern of intracellular organelles dissolves.

Next to functional interaction, the peroxisomal biogenesis process may also account for these observations. In adipocytes, peroxisomes are located in close proximity to lipid droplets for bidirectional substrate transfer, and under increased oleate concentrations, they even dock directly (Binns et al., 2006; Pu et al., 2011). This coordination between lipid droplets, peroxisomes and mitochondria was regulated by adipocyte triglyceride lipase and PPARA axis (Zhou et al., 2018). Furthermore, it has long been observed that the number of peroxisomes increases during adipocyte differentiation (Novikoff and Novikoff, 1982). It is tempting to speculate that comparable alterations might also be involved in the overall clearance of lipids, even in ectopic lipid accumulation in hepatocytes.

In line with this, the most prominent downstream alteration in the differential abundant peroxisomal proteomes is summarized under the key term “formation of peroxisomes.” The overrepresented data in alb-SREBP-1c contain vastly more interacting peroxisomal proteins, including 15 different PEX proteins, than in C57Bl6. However, the central hubs are PEX2, PEX3, PEX6, and PEX19. PEX19 is essential for both the import of peroxisomal membrane proteins and the *de novo* formation of peroxisomes. PEX19, in cooperation with PEX3, are the only proteins known so far to be necessary for the formation of peroxisomal membranes. However, a recent review discusses the finding of a general action of these proteins in organelle membrane sorting, including the ER, lipid droplets, and mitochondria (Jansen and van der Klei, 2019). PEX19 is anchored in peroxisome membranes. The farnesylation of PEX19 regulates its intracellular localization and may therefore be involved in selective interaction with different membranes (Agrawal et al., 2017; Emmanouilidis et al., 2017; Schrul and Schliebs, 2018).

The localization of peroxisomal membrane proteins, e.g., in mitochondria, was addressed by recent studies. It is clear that peroxisomes have a highly dynamic system of *de novo* genesis and division. However, the physiological regulation of peroxisome *de novo* biogenesis is still unclear. According to the conventional view, peroxisomes grow and divide from already existing organelles and can also develop *de novo* from the endoplasmic reticulum (Lazarow and Fujiki, 1985; Smith and Aitchison, 2013; Jansen and van der Klei, 2019). De nov*o* peroxisomal biogenesis does not solely involve peroxisomal budding from the ER. It has been shown that this is a more complex process, including the development of pre-peroxisomes originated from mitochondria, which fuse with endoplasmic vesicles from the ER for maturation by a PEX3 cycling process (Sugiura et al., 2017). On the other hand, the specific loss of hepatic peroxisomes, e.g., in liver-specific PEX5^–/–^ KO mice, leads to mitochondrial malformation or function in the liver but not in other physiological relevant tissues (Dirkx et al., 2005; Shinde et al., 2018; Tanaka et al., 2019). This indicates that the generation of peroxisomes is functionally closely related to the mitochondria. At the same time, this may be the basis for the observation of “classical” mitochondrial functions reported in diet-induced hepatic lipid accumulation (Krahmer et al., 2018). In line with this, an increased proportion, e.g., of proteins involved in oxidative phosphorylation, have been identified, especially in the alb-SREBP-1c model.

Another source of the increase in proteins in peroxisomes is import. All peroxisomal proteins are synthesized in cytosol and are imported into the organelles. Peroxisomes have a broad spectrum of import mechanisms. From the first observations of the peroxisomal signal peptides PST1 and PTS2, the post-translational import processes are now combined into a peroxisomal importomer and include proteins, post-translationally modified and folded proteins, or protein complexes (Walter and Erdmann, 2019). The peroxisomal translokon differs from import machines of other organelles such as mitochondria or ER because folded and even oligomeric proteins are able to cross the peroxisomal membrane without prior unfolding (Glover et al., 1994; Mcnew and Goodman, 1994; Walton et al., 1995). However, components of the peroxisomal import machine are initially located in ER and mitochondria and are combined during the fusion of the mitochondrial pre-peroxisomes with ER (Rai and Bhatnagar, 2017).

## Conclusion

In conclusion, genetically increased hepatic DNL is accompanied by marginal gene expression changes and changes in the amount of proteins in peroxisomal fractions. Based on these observations, it may be speculated that organelle plasticity may be altered to compensate for ectopic lipid accumulation. This might involve impaired or improper organelle biogenesis. Here, the coordinated interaction of the organelles, due to the altered PC- and lysoPC-species and resulting in alterations in membrane plasticity, could be a target to interfere with ectopic lipid accumulation.

## Data Availability Statement

The datasets generated for this study can be found in the full datasets are available under accession number GSE132298.

## Ethics Statement

The animal study was reviewed and approved by the Animal Care Committee of the University Duesseldorf approved animal care and procedures (Approval#84-02.04.2015.A424; 02 April 2015). Written informed consent for participation was not obtained from the owners because Mouse model was generated by the last author (Knebel et al., 2012).

## Author Contributions

BK and JK were responsible for experimental design, interpretation, writing and editing of the manuscript, and performed *in silico* analyses. SH, SL, and UK researched the proteomic data. MS and DH performed the lipidomic analyses. SJ, PF, MD, and NW researched data for metabolic characterization and gene expression. CK was responsible for animal care. DM-W contributed to experimental design, interpretation of data, review and editing of the manuscript. JK was the principal investigator of the study.

## Conflict of Interest

The authors declare that the research was conducted in the absence of any commercial or financial relationships that could be construed as a potential conflict of interest.

## Footnotes

1 www.ncbi.nlm.nih.gov/geo/

## Abbreviations

ALT: alanine transaminase
AST: aspartate transaminase
BG: blood glucose
DNL: *de novo* lipogenesis
EFA: essential fatty acids
ER: endoplasmatic reticulum
FFA: free fatty acids
GO: gene ontology
HOMA-%ß: Homeostatic model assessment of ß-cell function (%)
HOMA-IR: Homeostatic model assessment of insulin resistance
IR: insulin resistance
LysoPC: Lyso-phosphatidylcholine
MS: mass spectrometry
MUFA: monounsaturated fatty acids
NAFLD: non-alcoholic fatty liver disease
NEFA: non-saturated fatty acids
PC: phosphatidylcholine
PUFA: poly unsaturated fatty acids
SCD1: Δ 9 stearoyl-CoA desaturase 1
SFA: saturated fatty acids
SREBP: sterol regulatory element-binding protein
TFA: total fatty acids
TG: triglycerides
UFA: unsaturated fatty acids.
