# Supplementary material for: Fatty Liver Due to Increased de novo Lipogenesis: Alterations in the Hepatic Peroxisomal Proteome
Source: Front Cell Dev Biol. 2019 Oct 25;7:248. doi: 10.3389/fcell.2019.00248 (PMC6823594; doi:10.3389/fcell.2019.00248)
Supplement: Supplementary file 1 [file Presentation_1.zip › Table 11.DOCX]

**Supplementary Figures**

**
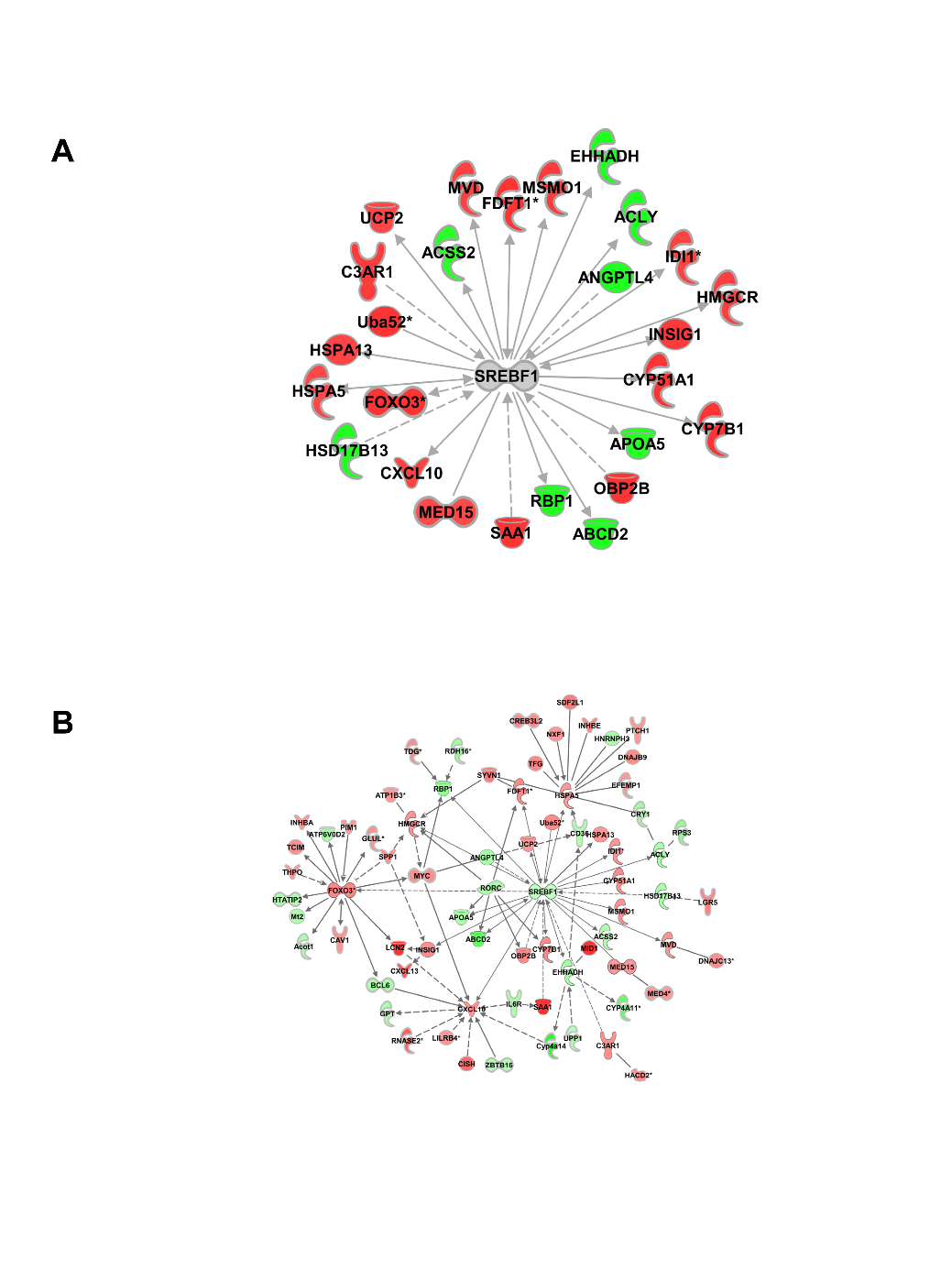
**

**Supplementary Figure S1** Holistic hepatic gene expression analyses C57Bl6 and alb-SREBP-1c mice identified 18 direct and 6 indirect downstream target genes of SREBP1 with 1.4-fold expression difference (A). (B) Expanded SREBP1 interaction network derived from gene expression analyses. Analyses were performed in IPA^TM^. Molecules: green: negative fold change (more abundant in C57Bl6); red: positive fold change (more abundant in alb-SREBP-1c).

**A**

|  | C57Bl6 | | alb-SREBP-1c | |  |
| --- | --- | --- | --- | --- | --- |
|  | **mean** | **SD** | **mean** | **SD** | **p-value** |
| Cpt1a | 6.301 | 2.563 | 3.460 | 1.957 | n.s. |
| Cpt2 | 6.330 | 2.087 | 0.589 | 0.672 | <0.001 |
| Acot2 | 0.019 | 0.008 | 0.003 | 0.003 | <0.0001 |
| Pex5 | 19.190 | 6.762 | 0.670 | 0.003 | <0.0001 |
| Pex6 | 6.090 | 2.288 | 1.434 | 1.271 | <0.0001 |
| Pex7 | 0.044 | 0.030 | 0.005 | 0.002 | <0.01 |
| HmgCoR | 1.281 | 0.834 | 16.190 | 2.225 | <0.0001 |
| Fas | 8.621 | 4.612 | 106.300 | 45.828 | <0.0001 |
| Scd1 | 373.900 | 159.519 | 2017.000 | 142.547 | <0.0001 |
| Fads1 | 45.540 | 8.181 | 119.100 | 24.937 | <0.001 |
| Fads2 | 20.980 | 7.095 | 82.780 | 16.896 | <0.0001 |
| Pklr | 58.830 | 6.950 | 90.590 | 11.996 | <0.001 |
| Me1 | 0.006 | 0.002 | 0.015 | 0.001 | <0.0001 |

**B**

|  | C57Bl6 | | alb-SREBP-1c | |  |
| --- | --- | --- | --- | --- | --- |
|  | **mean** | **SD** | **mean** | **SD** | **p-value** |
| Cpt1a | 1925.59 | 108.41 | 1758.46 | 105.10 | <0.01 |
| Cpt2 | 1929.86 | 125.12 | 1350.21 | 60.24 | <0.0001 |
| Acot2 | 207.05 | 29.54 | 140.37 | 20.75 | <0.001 |
| Pex5 | 459.65 | 23.48 | 310.76 | 31.83 | <0.0001 |
| Pex6 | 438.31 | 34.85 | 338.70 | 34.60 | <0.0001 |
| Pex7 | 560.29 | 81.29 | 443.45 | 71.21 | <0.01 |
| HmgCoR | 1144.37 | 193.05 | 1732.82 | 509.12 | <0.01 |
| Fas | 3109.56 | 255.16 | 4376.29 | 688.21 | <0.001 |
| Scd1 | 17833.46 | 1227.96 | 20728.79 | 2257.62 | <0.01 |
| Fads1 | 5171.58 | 435.64 | 6595.43 | 370.80 | <0.0001 |
| Fads2 | 5356.39 | 475.30 | 6389.90 | 300.33 | <0.001 |
| Pklr | 2066.21 | 213.02 | 2686.29 | 229.42 | <0.0001 |
| Me1 | 1967.83 | 282.75 | 2701.48 | 270.74 | <0.001 |

**Supplementary Figure S2. Hepatic gene expression of exemplified genes in C57Bl6 and alb-SREBP-1c mice.** (A) The hepatic expression level of genes were determined by RT-PCR (n= 20 each) from the cDNA equivalent of 20 ng total RNA with a gene specific hybridization probes (Assay on demand, Thermofischer Scientific, Darmstadt, Germany). The relative RNA amount in arbitrary units was calculated ± SD. (B) The values of the corresponding overall gene expression data derived from MouseGene 1.0 Arrays determined by TAC expression analyses (Applied Biosystems, Darmstadt, Germany)). P-value was determined by Student’s t-test.

Acot2, Acyl-CoA Thioesterase 2; Cpt, Carnitine Palmitoyltransferase; PEX, Peroxisomal Biogenesis Factor; FAS, fatty acid synthase; HMG-CoAR, 3-hydroxy-3-methyl-glutaryl-CoA reductase; Me-1, malic enzyme; PEPCK, phosphoenolpyruvate carboxykinase; Pklr, Pyruvate Kinase liver type; SCD, stearoyl-CoA desaturase.

.





**Supplementary Figure S3** Lipid compositions of C57Bl6 and alb-SREBP-1c at the age of 24 weeks (A) fractional composition of serum FFAs and %-change within C57Bl6 and alb-SREBP-1c mice and the sums, non-saturated FA (USAT), monounsaturated FA (MUFA), saturated FA (SFA), essential FA (EFA, cC18:2+cC18:3) or non-essential FA (NEFA, C16:0+cC16:1+C18:0+cC18:1). Data are expressed as mean ± SD (n = 15 of each genotype). *p < 0.05, **p < 0.01 by Student's t‐test.


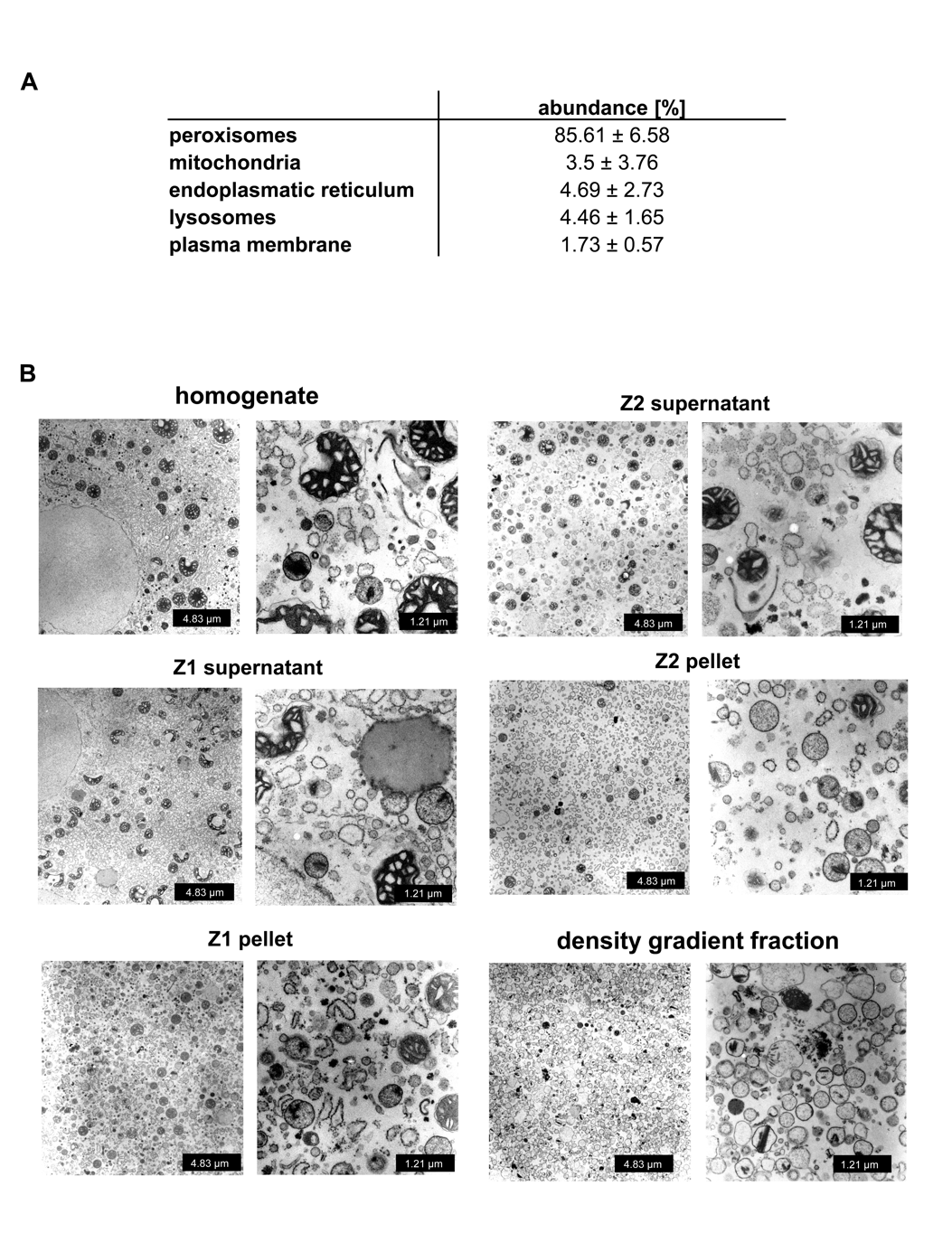


**Supplementary Figure S4. Enrichment of peroxisomes of a mouse liver via linear iodixanol gradient.** A freshly removed mouse liver was homogenized (homogenate) and the homogenate was centrifuged at 3,000x g (10min, 4°C). The supernatant (Z1) was then centrifuged at 17,000x g (15min, 4°C). The pellet (Z2) was placed on a linear iodixanol gradient (20-40%) and centrifuged at 100,000x g (80min, 4°C). The fractions with highest catalase activity were pooled and the peroxisomes were pelleted by centrifugation at 30.000x g (15min, 4°C). (A) Composition of peroxisome preparation calculated by marker enzyme activity**.** (B) A sample for electron microscope images was taken from each processing step.

**

**

**Supplementary Figure S5. Enrichment of peroxisomes of a mouse liver via linear iodixanol gradient.** Organellar composition of the peroxisomal fraction were monitored by assessing organelle specific marker enzymes according to the specified citations as follows:  catalase, peroxisomes (1), succinate dehydrogenase, mitochondria (2); alkaline phosphatase, plasma membrane; glucose-6-phosphatase, endoplasmic reticulum; acidic phosphatase, lysosomes (3).

1. Aebi, H. (1984) Catalase in vitro, Methods Enzymol. 105, 121−126.

2. Pennington, R. J. (1961) Biochemistry of dystrophic muscle. Mitochondrial succinate-tetrazolium reductase and adenosine triphosphatase, Biochem. J.80, 649−654.

3. Bergmeyer, H. U. (1974) Methoden der Enzymatischen Analyse, Verlag Chemie, Weinheim, Germany.
